# Synthesis and properties of a high-performance environment-friendly micro–nano filtration reducer

**DOI:** 10.1039/d0ra07504c

**Published:** 2020-11-27

**Authors:** Yanling Wang, Baoyang Jiang, Jincheng Lan, Ning Xu, Jinsheng Sun, Lingtao Meng

**Affiliations:** College of Petroleum Engineering, China University of Petroleum (East China) Qingdao 266580 China wangyl@upc.edu.cn

## Abstract

In this research study, we modified hydroxyethyl cellulose to obtain hydrophobically associating hydroxyethyl cellulose, and grafted it onto the surface of nano-calcium carbonate to obtain a graft copolymer. The intramolecular or intermolecular associations between the macromolecular chains of the graft copolymers form different forms of supramolecular network structures, and they interact with nanoparticles to form stable structures to enhance their related properties. The structure of the obtained graft copolymer was characterized by Fourier transform infrared spectroscopy (FT-IR) and laser particle size analysis. Thermogravimetric analysis (TGA) showed the thermal stability of the graft copolymer, and the results showed that the graft copolymer obtained thermally decomposed after 370.86 °C, indicating that it has good thermal stability. Scanning electron microscopy (SEM) revealed the mechanism of the graft copolymers in drilling fluids. The fluid loss control performance and rheology of the filtration reducer were evaluated before and after hot rolling at 180 °C for 16 hours. The results showed that the graft copolymer has excellent fluid loss reduction performance, and it has good fluid loss reduction performance in fresh water, brine and saturated brine. The API fluid loss was only 6.4 mL after hot rolling at 180 °C for 16 h in the brine base slurry. Moreover, the obtained graft copolymer is easily biodegradable, has EC_50_ ≥ 30 000 and good environmental performance, and can be used in high temperature and high salt reservoir with high environmental protection requirements.

## Abstract 2

In this research study, we modified hydroxyethyl cellulose to obtain hydrophobically associating hydroxyethyl cellulose, and grafted it onto the surface of nano-calcium carbonate to obtain a graft copolymer.

## Graphical abstract

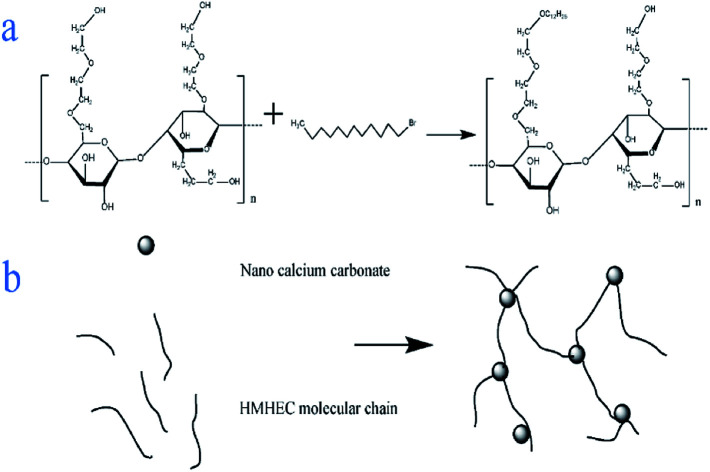

## 1. Introduction

With the continuous development of oil and gas field exploration in deep reservoirs, there are more deep and ultra-deep wells, and the demand for high-performance environmentally friendly drilling fluid treatment agents is more urgent. In recent years, people have paid more attention to the environmental protection performance of drilling fluids.^1^ Although filtration reducers for sulfonated and lignite drilling fluids have good temperature resistance, they cause irreversible damage to the reservoir due to their poor environmental protection. Under such drilling conditions, the coordination of the filterability and environmental protection of the drilling fluid has become more important. Thus, the research and application of high-performance environmentally friendly filter-reducing agents have received extensive attention. At present, many countries have made some progress in drilling fluid technology research, but they often fail to take into account environmental protection and drilling fluid performance. In addition, there are problems such as high cost and difficulty in field promotion.^2–4^ The high-performance environmentally friendly filtration reducer can take into account both environmental protection and drilling fluid performance.

Natural plants such as starch, xanthan gum, tannin, lignin, vegetable gum, and cellulose have the advantages of non-polluting and biodegradable properties. In addition, high molecular weight polymers based on natural plant synthesis have been widely used in petroleum exploration.^5^ In the past few decades, natural high molecular weight polymer filtration reducers have developed rapidly. According to previous studies, some modified starches have API fluid loss between 10 and 20 mL after aging at 150 °C.^6^ Saudi Arabia uses a filtrate reducer produced by the local plant date palm, which is suitable for freshwater and saltwater drilling fluids. It also has good environmental performance, and its HTHP filter loss is 30 mL after the filtrate reducer is added to the clay-free freshwater drilling fluid. Furthermore, its HTHP filter loss is 20 mL after the filtrate reducer is added to the clay-free seawater drilling fluid.^7^ A filtrate reducer synthesized from a low-viscosity polyanionic cellulose polymer is suitable for freshwater, saltwater and seawater conditions, can effectively control fluid loss, and has little effect on rheology.^8^

Although various cellulose-based graft copolymers have been synthesized and applied to water-based drilling fluids, the application of nanocomposite polymers synthesized by the introduction of nano-materials into cellulose-based polymers in water-based drilling fluids has been reported less.^9^ Cellulose has the advantages of wide distribution, high yield and easy biodegradation.^10,11^ Cellulose is alkali-treated from refined cotton and reacted with ethylene oxide as an etherifying agent in the presence of acetone to produce hydroxyethyl cellulose (HEC), which has strong water absorption and is a kind of nonionic cellulose.^6,12,13^ HEC can be modified by attaching a hydrophobic group to a hydroxyethyl group to obtain a hydrophobically modified HEC (HMHEC).^14,15^ The hydrophobic associating hydroxyethyl cellulose has a high aspect ratio, high specific surface area, good mechanical properties and biocompatibility,^16–20^ and is composed of a long-chain hydrophilic main chain, but its molecular structure contains a small amount of hydrophobic side chains or end groups.^21,22^ In addition, their skeleton structure will make the polymer dissolve in the aqueous medium. The intermolecular or intramolecular association of the hydrophobic groups also forms a reversible three-dimensional network structure. Furthermore, the intermolecular hydrophobic association region has high affinity, and has a significant impact on its performance.^15,23–25^ With the emergence of new nanomaterials, the application of polymer nanocomposites is rapidly developing. Due to the size effect, specific surface effect, selective adsorption and other properties of the nanoparticles,^26^ the introduction of nanoparticles into a single polymer matrix can enhance the relevant properties of the polymer and give it other excellent properties. This can reduce the application of other materials and save costs, and the biopolymer nanocomposites have better performance and environmental protection characteristics.^27^ Nanomaterials are used in water-based drilling fluids to improve the lubricity of drilling fluids, and to improve the rheology and fluid loss of drilling fluids.^11^ In this study, we proposed the branching of the short link with hydrophobic groups to the main chain of hydroxyethyl cellulose to obtain hydrophobic polymerized hydroxyethyl cellulose by carrying out a chain transfer reaction to the polymer. The hydrophobically associating hydroxyethyl cellulose is adsorbed on the nano calcium carbonate to form a micro–nano environmentally friendly filtration reducer.^28,29^

## 2. Experimental

### 2.1 Materials

When preparing a high-performance environmentally friendly filtration reducer, the reagents and materials used are: hydroxyethyl cellulose, which can be obtained from Feicheng Yutian Chemical Co., Ltd.; and 1-bromododecane, which was purchased from Macklin. The remaining chemical reagents were purchased from the Guo Yao Group Chemical Reagent Co., Ltd. All reagents and materials were stored at room temperature and under anhydrous conditions. The calcium-based bentonite used for the preparation of the drilling fluid base slurry was purchased from Weifang Huawei Bentonite Group Co., Ltd. China.

Preparation of base slurry: 0.8 g sodium carbonate was added to 400 mL water. Then, 16 g of bentonite was added on the high-speed mixer while stirring. The mixture was stirred continuously for 20 minutes, and attention was paid to scrape off the bentonite adhering to the wall of the cup during the stirring. The mixture then underwent static hydration for 24 h at room temperature.

### 2.2 Synthesis of silicone copolymer

**Fig. 1:**
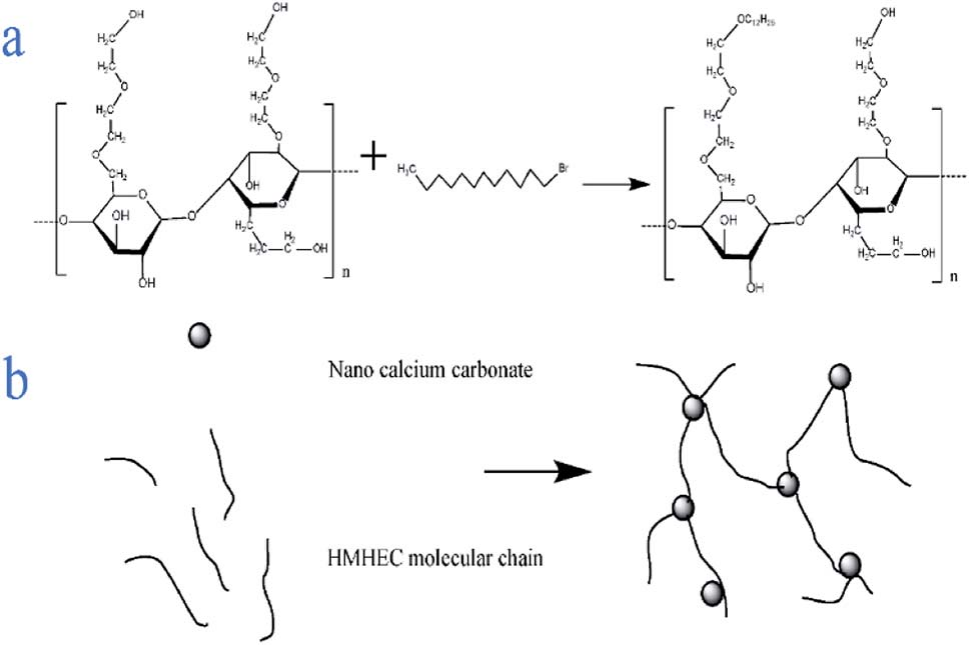
Synthesis of graft copolymer (a), and adsorption of nano calcium carbonate (b).

Solid sodium hydroxide (2.5 g) and absolute ethanol were put into a three-necked flask and magnetically stirred for 20 minutes to dissolve the mixture evenly. Nano-calcium carbonate (10 g) and 1-bromododecane were dissolved in a trace amount of absolute ethanol and added to the solution, magnetically stirred for 20 minutes, and then ultrasonically shaken for 30 minutes. The above suspension was heated to 65 °C, while magnetically stirring. After the temperature became constant, hydroxyethyl cellulose (10 g) was added and the mixture was stirred for 5 h. The liquid obtained above was then centrifuged at a high speed of 3000 rpm for 10 min to get the lower solid. The obtained solid was put into a thermostat at 80 °C for drying and grinding to obtain a hydroxyethyl cellulose graft copolymer (Fig. 1).

### 2.3 Characterization of filtration reducer

**Fig. 2:**
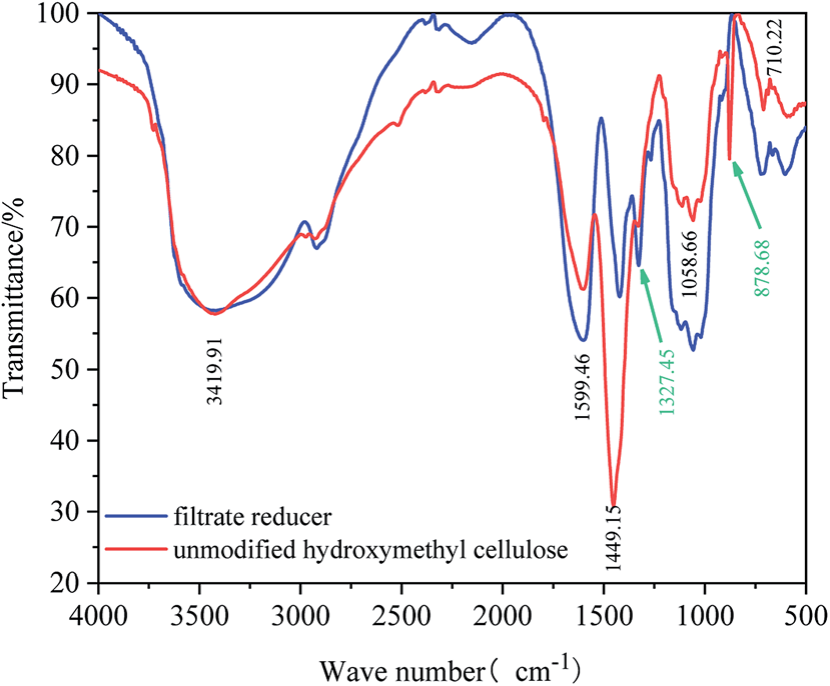
FTIR spectral analysis of filtrate reducer.

**Fig. 3:**
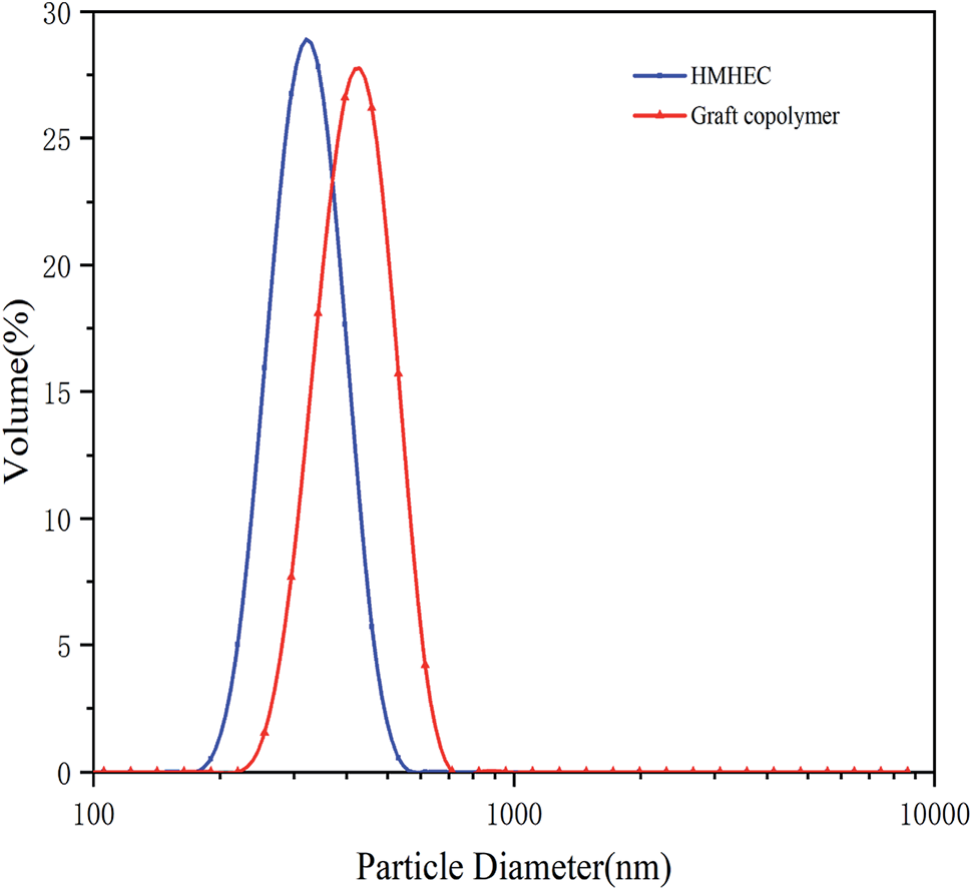
The particle size distribution curve of the hydrophobically modified hydroxyethyl cellulose and filtrate reducer.

**Fig. 4:**
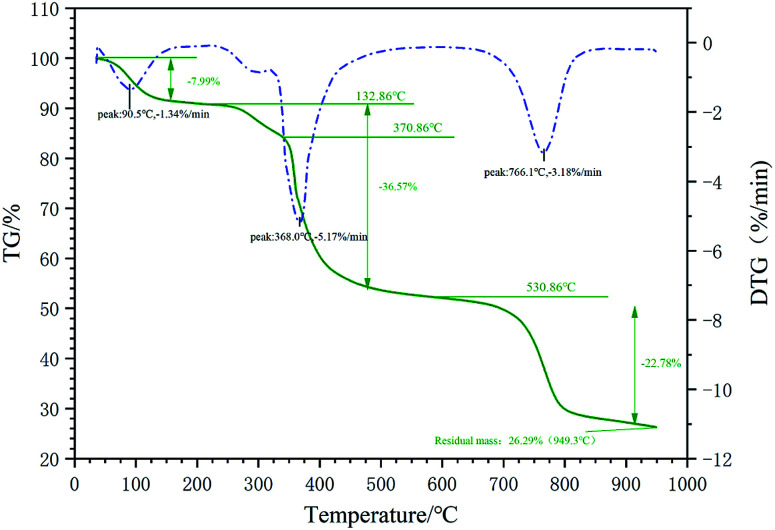
TGA of the filtrate reducer.

The particle size distribution measurement of the micro–nano particles was obtained by the Zetasizer Nano instrument, which can determine the particle size in the range of 15 nm to 500 μm by laser diffraction method. Before testing, the test product was put into a 0.1% aqueous solution, and then it was configured. The aqueous solution was then placed in the sample cell for laser particle size scanning (Fig. 2). A laser particle size analyzer was used to characterize the infrared spectroscopy. A small amount of filtrate reducer monomer was placed in a mortar, then uniformly ground with potassium bromide, and compressed with a tablet press. Scanning the filtrate reducer monomer in the wavelength range of 500 cm^−1^ to 4000 cm^−1^ to obtain its infrared spectrum (Fig. 3). A thermal analyzer (TGA) was used to measure the thermal stability of the filtrate reducer in a nitrogen atmosphere, with a heating rate of 10 °C min^−1^ and a temperature range of 35 °C to 950 °C (Fig. 4). A Microtox type biological toxicity tester was used to measure the biological toxicity of the fluid loss control agent. Table 1 shows the environmental evaluation standards of environmentally friendly drilling fluid treatment agents.

**Table 1:** Environmental evaluation standards for environmentally friendly drilling fluid treatment agents

Project	Standard value
Biological toxicity EC_50_, mg L^−1^	≥30 000
Biodegradability BOD_5_/COD_cr_, %	≥10.0

### 2.4 Characterization of the microscopic morphology of the filter cake

The medium pressure filter loss instrument (Kence Instrument (Shanghai) Co., Ltd.) was used to measure the fluid loss volume of the base pulp before and after aging at 160 °C/16 h. The obtained filter cake was dried naturally, and then gold was sprayed on the filter cake. Then, an electron microscope, JSM 7900F, (Japan Electronics Co., Ltd.) was used to scan the filter cake.

### 2.5 Rheological and fluid loss properties of drilling fluid with filtrate reducer

The rheology of the drilling fluid added with the filtration reducer was tested using a MOD ZNN-D6 six-speed rotary viscometer (Qingdao Haitongda Special Instrument Co., Ltd.). Its apparent viscosity (AV), plastic viscosity (PV) and yield point (YP) were measured according to API standards. Under the conditions of temperature of 25.0 °C and pressure of 0.69 ± 0.03 MPa, a SD3B triple medium pressure filter loss instrument (Kense Instrument (Shanghai) Co., Ltd.) was used to measure the static filter loss (FL_API_). The GGS42 type high temperature and high-pressure filter device (Qingdao Jiaonan Tongchun Machinery Petroleum Instrument, China) was used to measure the filtration loss (HT_HPAPI_) of HTHP at a pressure of 4.2 ± 0.03 MPa and a temperature of 180.0 °C. The measurement was performed within 30.0 minutes, according to API standards. The rheological parameters were calculated as follows:^30,31^AV = *θ*600/2 (mPa s)PV = *θ*600 − *θ*300 (mPa s)YP = (*θ*300 − PV)/2 (Pa)

**Fig. 5:**
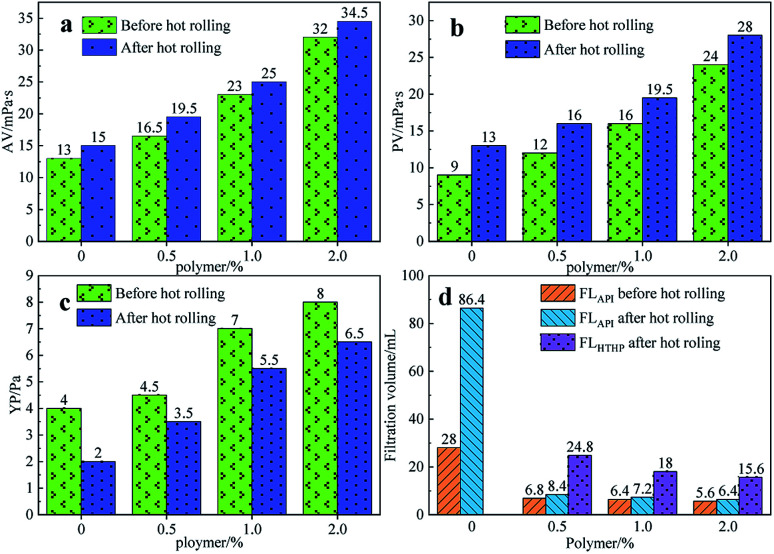
The effect of the filtrate reducer addition on AV (a), PV (b), YP (c) and FL (d) before and after hot rolling at 180 °C for 16 h.

In order to study the rheology and fluid loss properties of the filtrate reducers, different mass fractions (0%, 0.5%, 1%, 2%) of filtrate reducers were added to the 4% fresh water base slurry and stirred at high speed for 20 minutes. The samples were then heated at 180 °C for 16 hours. Then, its medium pressure filter loss, high temperature and high-pressure filter loss and various rheological parameters, and the results are shown in Fig. 5.

### 2.6 Biodegradability and biological toxicity

#### 2.6.1. Biological toxicity

Biodegradability refers to the possibility of environmental pollutants being degraded by microorganisms. It is an important indicator for evaluating the acceptance of organic matter by the environment. At present, the commonly used evaluation method at home and abroad is the BOD_5_/COD_cr_ ratio evaluation method. The biodegradability of organic matter can be evaluated by the BOD_5_/COD_cr_ ratio. The higher the ratio, the easier it is to be biodegraded.

The biodegradability of the fluid loss control agent was analyzed by measuring the biochemical oxygen demand, the BOD_5_ and chemical oxygen demand COD_cr_ of the decomposed fluid loss control agent, and the BOD_5_/COD_cr_ ratio was then calculated. The chemical oxygen demand COD_cr_ is the amount of oxidant consumed to oxidize the reducing substances in 1 L of water sample. 20 mL of 1% filtrate reducer aqueous solution was oxidized by adding 10 mL of potassium dichromate with a concentration of 0.25 mol L^−1^, and then titrated with (NH_4_)Fe(SO_4_)_2_ to calculate the chemical oxygen demand of the fluid loss agent.

The biochemical oxygen demand BOD_5_ was collected and domesticated by surrounding bacteria after adding a certain amount of filtrate reducer solution was added, incubated for 5 days and measured by BOD_5_ tester.

#### 2.6.2. Biodegradability

Testing the EC_50_ value of the biological toxicity of the drilling fluid with the fluid loss control agent, this experiment used the luminescent bacteria method to determine the biological toxicity of the drilling fluid, and used EC_50_ (when the relative luminescence rate is 50%) to characterize the biological toxicity of the test object. The larger the EC_50_ value, the lower the biological toxicity of the test substance; the smaller the EC_50_ value, the greater the biological toxicity of the test substance.

## 3. Results and discussion

### 3.1 Characterization of filtrate reducer

#### 3.1.1. Infrared spectroscopy characterization

As shown in Fig. 2, a comparative analysis of the FTIR spectrum of the fluid loss agent and hydroxyethyl cellulose shows that the tensile vibration of the –OH group in the cellulose molecule is represented by a peak at 3419.91 cm^−1^. The peak at 1599 cm^−1^ represents the C=C tensile vibration in the cellulose molecule. There is no peak position between the peaks of 1449 cm^−1^ and 1058 cm^−1^ in the FTIR spectrum of hydroxyethyl cellulose. Compared with the FTIR spectrum of the fluid loss agent, the peak position has decreased to 1327.45 cm^−1^ for the reactive –OH bending vibration. The analysis shows that the more reactive hydroxyl group on the hydroxyethyl cellulose molecular chain undergoes a Williamson etherification reaction with halogenated alkanes,^32^ and the grafting reaction introduces long-chain alkanes into the hydrophilic molecular chain of hydroxyethyl cellulose. In addition, the infrared spectrum of the filtrate reducer increased the peak at 878.68 cm^−1^. The bending vibration at 878.68 cm^−1^ is formed by the long chain of the introduced alkane. It can be seen from the infrared spectrum of the copolymer that the copolymer contains all of the molecular groups originally designed, which indicates that the hydroxyethyl fiber bundle and 1-bromododecane has formed a graft copolymer.

#### 3.1.2. Particle size analysis

Fig. 3 shows the particle size distribution curve of the hydrophobically modified hydroxyethyl cellulose and filtrate reducer. The average particle size of the filtrate reducer is slightly larger than that of the hydrophobically modified hydroxyethyl cellulose, indicating that the modified hydroxyethyl cellulose is successfully adsorbed on the nano calcium carbonate particles. It can be seen from Fig. 3 that the particle size of the graft copolymer is still in the nanometer domain, and still good for plugging and reducing fluid loss.^33,34^

#### 3.1.3. Thermal stability of the filtrate reducer

As shown in Fig. 4, when the temperature range is 35 °C to 132.86 °C, the weight loss rate at this stage is relatively fast, and the weight loss rate is basically unchanged for a long period of temperature range thereafter. This is due to the presence of a certain number of hydrophilic groups in the molecular structure of the graft copolymer, which makes the measured sample absorb part of the free water in the air, and the faster rate of weight loss is caused by the volatilization of this part of the water. In the temperature range of 132.86 °C to 370.86 °C, the weight loss rate at this stage slows down. This is because the stronger molecular groups in the molecule interact with the water molecules in the environment and form strongly adsorbed bound water on the groups. The volatilization of bound water is slower than that of free water before, so the rate of weight loss is also slower. In the temperature range of 370.86 °C to 530.86 °C, the thermal weight loss rate is very fast at this stage, and groups such as hydroxyl and ether groups begin to decompose rapidly at this stage. When the temperature is between 530.86 °C and 949.3 °C, the main chain and side chains of the copolymer begin to break. With the endothermic process, the quality of the copolymer begins to continue to decline. In addition, nano-calcium carbonate begins to thermally decompose at about 600 °C, and nano-calcium carbonate is converted into CaO and CO_2_. The nano-calcium carbonate begins to decompose rapidly at about 800 °C. Most of the mass residues at 949 °C are CaO solids and a very small amount of nano-calcium carbonate that has not been completely decomposed. When the temperature reaches 949.3 °C, the mass retention rate is 26.29%. In summary, the thermogravimetric curve shows that the filtrate reducer has good thermal stability at high temperatures.

### 3.2 Rheology and fluid loss reduction of the filtrate reducer

Fig. 5 shows the effect of the filtrate reducer addition on the rheology and fluid loss control properties of freshwater-based drilling fluids. As the temperature rises, AV (a), PV (b) and YP (c) all increase. This is formed by several factors. In the first aspect, in the drilling fluid, the hydrophobic groups of the polymer are associated with each other. The intramolecular or intermolecular association occurs between the macromolecular chains, forming different forms of the micellar nanostructure-supramolecular network structure. In a dilute solution, the graft copolymer occurs mainly *via* intramolecular association, in which the macromolecular chain curls, the hydrodynamic volume decreases, and the intrinsic viscosity decreases. When the graft copolymer concentration exceeds a certain value, the macromolecular chain will form a dynamic physical cross-linked network structure dominated by intermolecular association, and the solution viscosity will be greatly increased. The second aspect is different from the irreversible shear degradation of general high molecular weight polymers. Under the action of a higher shear force, the cross-linked structure formed by the hydrophobic association of the graft copolymer is destroyed and the solution viscosity decreases. When the shearing effect is eliminated, the cross-linked network structure formed by the association between the macromolecules is reformed and the viscosity is restored. On the other hand, in addition to the van der Waals force, hydrogen bonding force and electrostatic force between HMHEC and nano calcium carbonate particles, hydrophobic association also plays a major role. HMHEC is adsorbed on the surface of nano-calcium carbonate and forms a larger spatial network structure with it using the bridging mechanism, which improves the degree of cross-linking of the entire inorganic/organic system. Moreover, the hydrophobic association is an endothermic effect.^35^ In summary, the filtrate reducer has a certain temperature resistance and viscosity increase.

**Fig. 6:**
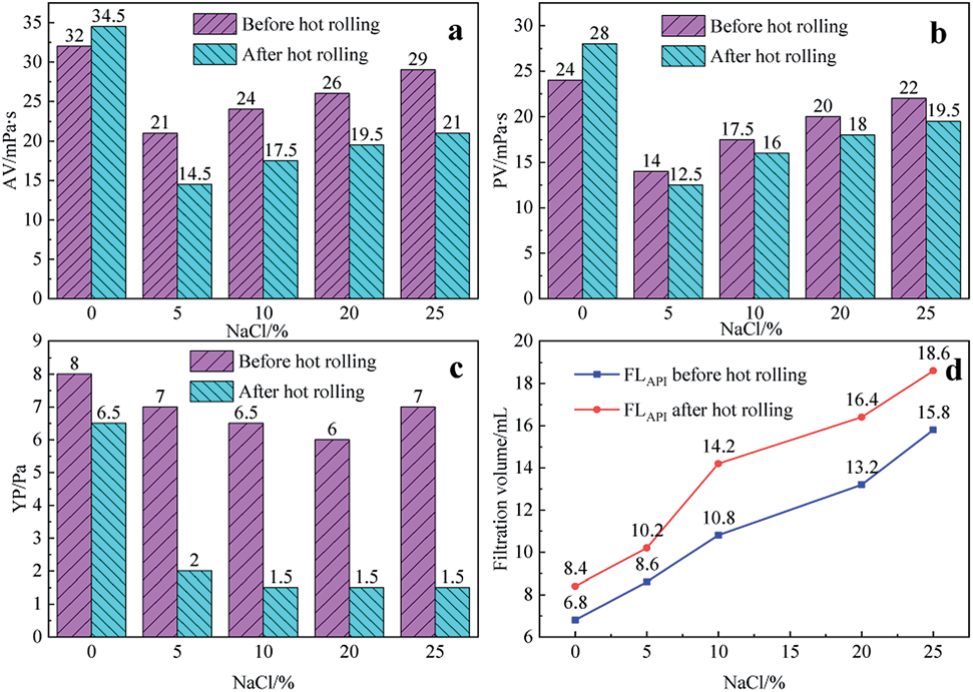
The effect of NaCl addition on AV (a), PV (b), YP (c) and FL (d) before and after hot rolling at 180 °C for 16 h.

**Fig. 7:**
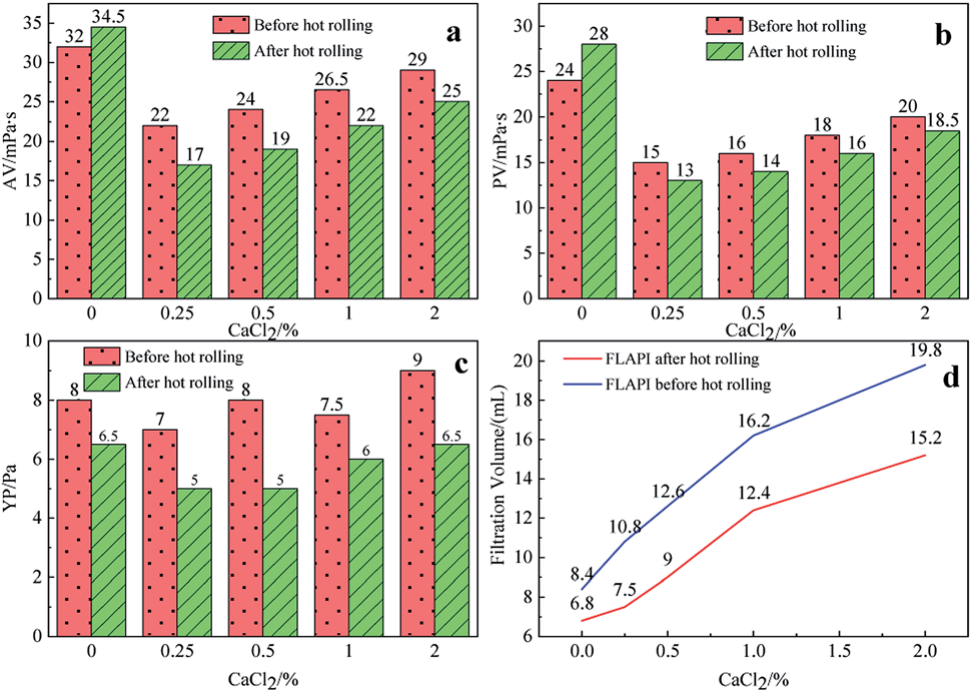
The effect of CaCl_2_ addition on AV (a), PV (b), YP (c) and FL (d) before and after hot rolling at 180 °C for 16 h.

**Fig. 8:**
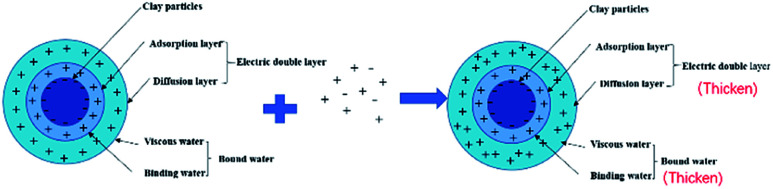
Schematic diagram of the change of the diffusion double layer after adding salt.

It can be seen from Fig. 6 and 7 that after 180 °C aging and increased added salt concentration the viscosity first decreases and then increases to a certain extent, and the fluid loss reduction increases. The viscosity is lower after adding salt than the condition without the added salt. This is because the added salt compresses the diffusion electric double layer of the clay particles. This makes the hydration film on the surface of the clay particles thinner and reduces the zeta potential between the clay particles, thereby reducing the repulsive force between the particles. This makes it easier for the clay particles to coalesce together, resulting in a decrease in the viscosity of the drilling fluid system. Later, as the amount of salt increased, the viscosity increased slightly. The reason for the analysis was that the addition of the small-molecule salt electrolytes would increase the polarity of the solution and increase the hydrophobic association between macromolecules. In addition, the macromolecular long chain of the graft copolymer can prevent the collision and coalescence between the clay particles to a certain degree, weaken the influence of the salt on the clay particles, and reduce the coalescence phenomenon of the clay particles. At the same time, as shown in Fig. 8, the hydrated group on the long chain of the macromolecule will increase the thickness of the clay particle diffusion electric double layer, and help to form a thinner and lower permeability filter cake.^36,37^ In addition, the electron pair on the carbonyl group on the graft copolymer long chain easily adsorbs calcium ions, reducing the influence of the calcium ions on the clay particles, thereby enhancing the stability of the drilling fluid system. However, as the amount of salt increased, the fluid loss of the drilling fluid did not increase significantly after aging at 180 °C. This is because the copolymer macromolecular chain can effectively shield the influence of salt on the clay particles, protect the clay particles from aggregation, help improve the stability of the drilling fluid system, and reduce the fluid loss under high salt conditions. In summary, the filtrate reducer has obvious salt and calcium resistance.

### 3.3 Microscopic morphology of drilling fluid filter cake

**Fig. 9:**
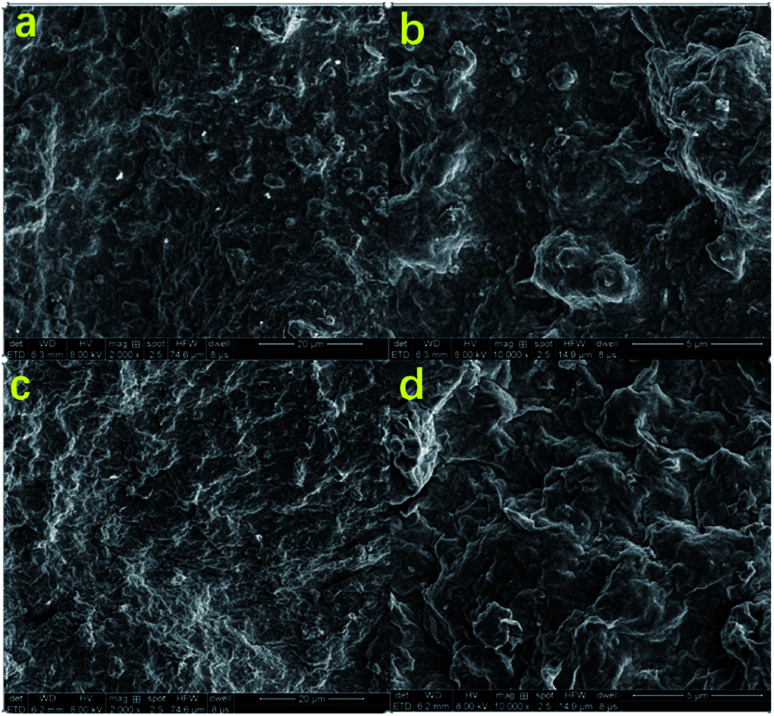
SEM images of the basic fluid cake (a and b), and the basic fluid cake (c and d) after adding 2% filtration reducer.

As shown in Fig. 9(a) and (b), the mud cake formed by high temperature aging at 180 °C for 16 hours without filtration reducer has a rough surface and large clay particle aggregate. In addition, the clay particles have very irregular shapes. There are some tiny pores and cracks on the surface of the mud cake. The clay cannot be fully hydrated to form a hydration film, which makes it easier for water to filter through the mud cake, resulting in excessive fluid loss. As shown in Fig. 9(c) and (d), the surface of the mud cake formed with the filtration reducer is smooth and compact. In addition, there is no large unevenness and no large clay particles are formed. This indicates that the fluid loss agent inhibits the coalescence of the clay particles and maintains the clay particles. The dispersibility makes the mud cake have excellent performance, and it is difficult for water to enter the formation, which improves the performance of reducing fluid loss. The active hydroxyl group in the modified hydroxyethyl cellulose molecule of the filtrate reducer can form hydrogen bonds with the Si–OH and Al–OH in the bentonite, further blocking the coalescence of clay particles. The filtration reducer has a small volume, large specific surface area, and many surface-active hydroxyl groups. It can form a spatial network structure connected by hydrogen bonds and van der Waals forces. The strength of this kind of space structure is limited, and it is destroyed under shearing action, but the rate of its reformation is also very fast. With the increase of the shear rate, the destruction and formation of the space grid structure become a dynamic balance. This reflects the excellent shear thinning. It can effectively block the filter cake and micro–nano voids, and play a role in reducing filtration. In addition, filtration reducers are micro–nano particles, and their higher specific surface area contributes more to heat.^38–40^ On the other hand, the fluid loss additive is a macromolecular copolymer, which has a certain viscosity increase and dynamic shearing effect in the drilling fluid base slurry. It can reduce the fluid loss to a certain extent, and the macromolecular polymer filtration reducer can also block the smaller pores in the drilling fluid, which can effectively reduce the fluid loss.^41–43^

### 3.4 Environmental performance evaluation

Testing the biodegradability of the drilling fluid base slurry added with a fluid loss agent, BOD_5_/COD_cr_ can indicate the biodegradability of the fluid loss agent. If BOD_5_ represents the non-biodegradable part of COD_cr_, the proportion of the non-biodegradable organic matter in the base pulp can be represented by BOD_5_/COD_cr_. When the ratio exceeds 0.45, it means that the non-biodegradable organic matter accounts for less than 20% of all organic matter. When the ratio is lower than 0.2, the non-biodegradable organic matter accounts for more than 60% of all organic matter. Using the dichromate method to test the value of COD_cr_. The determination of BOD_5_ adopts the GB7488-87 water quality five-day biochemical oxygen demand determination method.^44^

To test the EC_50_ value of the biological toxicity of the drilling fluid base slurry with added fluid loss agent, this experiment uses the luminescent bacteria method to test the biological toxicity of the fluid loss agent. Using EC_50_ (when the relative luminescence rate is 50%) to characterize the biological toxicity of the test substance, we found that the biological toxicity of the test substance decreased with larger EC_50_ value. In addition, a greater biological toxicity of the test substance resulted in a smaller EC_50_ value.^45^

**Fig. 10:**
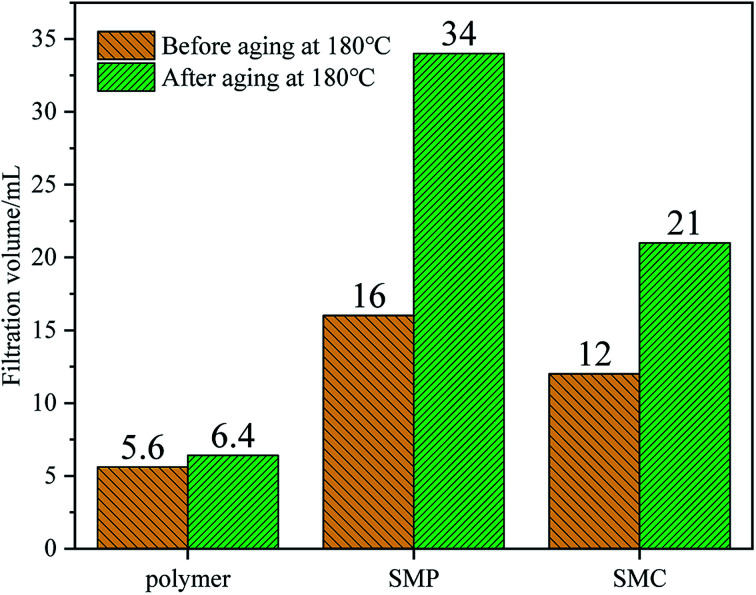
Effect of different filtrate reducers on the filtration failure in drilling fluids.

After the environmental performance test of the filtration reducer, the filtration reducer has *Y* = BOD_5_/COD_cr_ = 17.42%, which is a type that is easily biodegradable. A Microtox-type biological toxicity tester was used to test the biological toxicity of the filtrate reducer. The EC_50_ value of the filtrate reducer was 3.41 × 10^4^ mg L^−1^, indicating that the filtrate reducer was non-toxic (Fig. 10).

### 3.5 Comparison with other fluid loss additives

We compared the synthetic fluid loss agent with sulfonated lignite (SMC)^46^ and sulfonated phenolic resin (SMP),^47^ which are commonly used in oil fields. It can be seen that the fluid loss of the micro–nano environment-friendly fluid loss control agent synthesized in this paper before aging at 180 °C is 5.6 mL, and the fluid loss after aging at 180 °C is 6.4 mL. The micro–nano environment-friendly fluid loss reducer synthesized in this paper is better than other similar products.

## 4. Conclusion

In this paper, the hydrophobically associating hydroxyethyl cellulose was obtained by modifying the hydroxyethyl cellulose, and the graft copolymer was obtained by grafting it onto the surface of the nanometer calcium carbonate. Finally, the micro–nano environment-friendly filtration reducer was successfully synthesized. The structure of the filtration reducer was characterized by particle size analysis and infrared spectroscopy, and the mud cake was characterized by scanning electron microscope SEM. Its thermal stability was characterized by TGA, and the results showed that the filtration reducer has strong thermal stability. This is because the synthetic filtration reducer belongs to the micro–nano level, has a high specific surface area, and its contribution to contrast heat is higher. Moreover, the filtration reducer is a macromolecular copolymer, which has the effect of increasing the viscosity and dynamic shear force in the drilling fluid base slurry, and can reduce the fluid loss to a certain extent. In addition, the macromolecular polymer filtration reducer can block the micro–nano pore size in the drilling fluid by itself, stabilize the well wall, and can effectively reduce the fluid loss. The results showed that when the added amount of filtration reducer was 2.0 wt%, the fluid loss could be reduced to 6.4 mL after aging at 180 °C for 16 h. The results show that the filtration reducer has good thermal stability, salt and calcium resistance. In addition, from the scanning electron micrograph analysis of the mud cake, it can be seen that the surface of the mud cake formed by adding the filtration reducer to the base slurry is smooth and compact, which indicates that the filtration reducer inhibits the coalescence of the clay particles and maintains the dispersion of the clay particles. This makes it more difficult for water to enter the formation, and improves the performance of reducing fluid loss. Finally, through its environmental performance test, the results show that the filtration reducer has no biological toxicity and is easily biodegradable.

## Conflicts of interest

There is no conflict to declare.

## Supplementary Material
